# Perceptions of education quality and influence of language barrier: graduation survey of international medical students at four universities in China

**DOI:** 10.1186/s12909-020-02340-w

**Published:** 2020-11-07

**Authors:** Wen Li, Chang Liu, Shenjun Liu, Xin Zhang, Rong-gen Shi, Hailan Jiang, Yi Ling, Hong Sun

**Affiliations:** 1 School of International Education, Xuzhou Medical University, No.209 of Tongshan Road, Yunlong District, Xuzhou, Jiangsu China; 2 School of literature, Dali University, Dali, China; 3 College of International Studies, Nanjing Medical University, Nanjing, China; 4 School of International Education, Nanjing Medical University, Nanjing, China; 5 Department of Obstetrics and Gynaecology, First Affiliated Hospital of Hainan Medical University, Haikou, China

**Keywords:** Africa, Asia, Curriculum evaluation, Graduation questionnaire, International medical student, Language barrier, Medical education in China, Students’ perceptions

## Abstract

**Background:**

As the number of Asian and African students studying medicine in China increases, it is imperative to evaluate the educational experiences of these international medical students (IMSs). This study was intended to investigate opinions of China-educated IMSs towards the medical curriculum and the impact of Chinese language capability on their clinical studies.

**Methods:**

A self-administered questionnaire was circulated to the final-year IMSs during the graduation time from May 2019 to July 2019 in 4 universities in China. The questionnaire asked IMSs to assess the quality of medical education and provide a self-evaluation of their Chinese language capability. One-way Analysis of Variance (ANOVA) was used to determine whether IMSs’ Chinese language capability was associated with their clinical experiences and clinical competence.

**Results:**

Overall, we received 209 valid responses, of which 76.1% were satisfied with the quality of medical education. Genetics, physics, and mathematics were perceived as the least relevant basic courses for medical practice, and 21.5% of student reported that community-oriented medicine was a neglected subject. Notably, 58.9% of students had positive views about discussions on ethical topics during their clerkships, and 71.3% believed they had acquired sufficient clinical skills to begin a residency program. Chinese speaking skills and communication initiatives were found to be critical factors in influencing students’ clinical experiences and competence.

**Conclusion:**

This study presents the perceptions of China-educated IMSs towards medical curriculum from various aspects. Results show that language influences the education experiences of IMSs. Collectively, these results indicate that the curriculum for IMSs in China should be more problem-based and community-engaged to improve IMSs’ learning experiences and preparation for community deployment. Furthermore, training curriculum for the oral Chinese should be improved to equip IMSs with sufficient language competence to enable them to efficiently carry out clinical clerkship and rotations. Our findings provide evidence for benchmarking medical curricular codifications tailored for Asian and African students.

**Supplementary Information:**

The online version contains supplementary material available at 10.1186/s12909-020-02340-w.

## Background

In the last two decades, China has experienced a dramatic rise in the enrolment of international students to its institutions of higher education, and clinical medicine is the most-chosen program for academic studies [1]. A significant number of Chinese medical schools offer a 6-year English-taught undergraduate program for international medical students (IMSs), who are conferred with a bachelor of medicine degree on successful completion of their studies. The curriculum of this program for IMSs is similar in almost all universities and colleges. The 1st year study (preparatory year) encompasses liberal arts and natural science courses, and this is followed by 2 years (preclinical years) of basic medical science courses. In the 4th and 5th years of study, clinical courses integrated with bedside trainings are taught, while in the 6th year the students are subject to rotational internships. The 4th, 5th and 6th years are also referred to as the clinical years. IMSs are required to accomplish the compulsory courses and a certain number of optional courses in order to achieve the minimum credits based on the training plan of each university.

For effective communication, theoretical classes for IMSs are conducted in English. The curriculum for IMSs is derived from the curriculum for Chinese medical students, and is also tailored to cover the disease spectrum in the IMSs’ country of origin. During the internships, IMSs work together with Chinese students and, therefore, become internship partners. This approach helps foster a cooperative learning relationship between domestic and international students.

To produce internationally competent medical professionals, China trains IMSs in compliance with Global Minimum Essential Requirements in Medical Education [2]. A series of actions have also been taken to upgrade training standards. China International Medical Education Committee has been established [3], and national policies have been formulated [4–6] to develop and standardize international medical education.

Currently, over 68,000 IMSs are pursuing their studies in China, and a majority of them come from lower income countries of Asia and Africa [7]. These students constitute a significant number of potential medical workforce for both their home countries and the countries they may intend to migrate to [8, 9]. In light of the significant role played by these IMSs in the healthcare service of their future practice locations, it is important to assess the quality of international medical education in China.

Students’ feedback serves as an indispensable indicator for accessing the quality of medical education [10], and the graduation occasion has been proposed as an appropriate timing for collecting students’ views [11]. A number of studies conducted surveys on graduating students to reflect various aspects of healthcare profession education, exemplified by the assessments of medical education in the UK [12] and Vietnam [13], nursing education in the USA [14], and dental education in India [15].

The Association of American Medical Colleges (AAMC) has been administering the Graduation Survey (GQ) annually since 1978 to gather opinions from final-year medical students in the USA regarding their learning experiences and future plans [16], which proves to be an effective tool for identifying areas of strength and weakness in the medical program [17]. Developed from the version of the USA, the Canadian GQ is also applied nationally to evaluate the undergraduate MD programs in Canada [18, 19]. Due to the great longitudinal stability of the AAMC GQ [20], it has been utilized by researchers to evaluate the quality of medical education in many other countries as well, such as Nepal [21], Iran [22] and Israel [23]. There are also two studies from China, one conducted in mainland China to explore graduates’ perceptions on a medical school’s traditional and reformed curricula [24] and the other conducted in Taiwan in 4 medical schools to obtain graduates’ views on medical education [10].

However, to the best of our knowledge, there is no such comprehensive graduation survey directed at IMSs in China. Their perceptions on educational experiences deserve more attention. It is important to evaluate this medical program and obtain a feedback from these IMSs, based on their pre-college experiences and their future career expectations. This feedback is pivotal for curriculum improvement. Although the lectures are taught in English, the use of Chinese language is inevitable in the clinical environment, as most of the patients could only speak Chinese. The impact of this factor on IMSs’ clerkship and internship also needs evaluation.

The aim of this study was to evaluate IMSs’ perceptions of the international medical education in China and to identify the relationship between IMSs’ language capability and their clinical studies. The outcome of this study will provide basis for aligning medical education to suit the needs of IMSs from Asian and African countries.

## Methods

### Setting and participants

A questionnaire for IMSs (IMS GQ) was administered to the final-year IMSs during the graduation time from 31st May, 2019 to 1st July, 2019 in 4 universities in China. These universities are located in Xuzhou (east of China, Jiangsu Province), Dali (southwest of China, Yunnan Province), Nanjing (east of China, capital of Jiangsu Province) and Haikou (south of China, capital of Hainan Province). With the survey being performed in different cities with different economic and educational characteristics, we aim to present a more representative picture of international medical education in China.

### Questionnaire design

A self-administered questionnaire was developed by referencing AAMC GQ [16] and other graduation surveys [10, 18, 21–23]. A pilot study was conducted, the results from the pilot work were discussed by the team members, and the final version of the questionnaire, IMS GQ, was then formulated. This IMS GQ contained three sections. The first section collected students’ basic personal information, such as gender, age, nationality and university.

The second section asked about students’ language capability, including their self-evaluation of Chinese speaking skills, initiative to communicate in Chinese and information on obtaining the Hanyu Shuiping Kaoshi (HSK) certificate. HSK is a Chinese language proficiency test, a national written exam to test the candidates’ command of Chinese in listening, reading and writing. It is administered by the Confucius Institute Headquarters (Hanban), a subdivision of the Ministry of Education of China. HSK tests are classified into 6 levels, from the easiest to the most difficult.

The third section was comprised of 34 Likert-scale-type questions and 1 question enquiring about the location of the internship. These 35 questions were classified into 6 aspects, namely overall satisfaction (Q11, 1 item), basic science education (Q12-Q18, 7 items), clinical education (Q19-Q31, 13 items), clinical competence (Q32-Q38, 7 items), benefits of diversity (Q39-Q40, 2 items), and study hours for specific topics (Q41-Q45, 5 items).

### Data collection and analysis

In this study, electronic questionnaire was employed, except for University A, where a proportion of the respondents filled a paper version of the questionnaire. Each of the 4 participating universities had its own questionnaire distributor(s), who were administrators and academics involved in education for IMSs in the respective universities. At the beginning of the questionnaire, it was clearly stated that participation was voluntary and that the results could be used for research on medical education. All the participants gave informed consent for their opinions to be published anonymously.

To rate the extent to which participants agreed or disagreed with various statements, responses ranged from “strongly disagree (1 point)” to “strongly agree (5 points)”; to rate the quality of the basic and clinical courses, responses ranged from “very poor (1 point)” to “excellent (5 points)”; and to rate study hours for specific topics, responses ranged from “absent (1 point)” to “excessive (4 points)”. For discussion purposes, “strongly disagree/very poor/absent” and “disagree/poor/inadequate” were regarded as negative responses, while “strongly agree/excellent/adequate” and “agree/good/excessive” were regarded as positive responses. “Neutral/Fair” responses were omitted.

According to the proficiency level attained in the HSK tests described on the HSK official website [25], students who held a level 3 HSK certificate or below were categorized into the lower HSK level group, and those who held a level 4 HSK certificate or above were categorized into the higher HSK level group.

The data obtained through this study were analysed using IBM SPSS Statistics (version 24.0). The responses in the third section of the questionnaire were given as percentages. One-way Analysis of Variance (ANOVA) was used to determine whether respondents’ HSK levels, their Chinese speaking skills and their initiative for communicating in Chinese were associated with their clinical experiences and clinical competence. Figures were drawn using GraphPad Prism (version 8.4).

## Results

A total of 280 IMSs were randomly selected from the 4 universities to complete the IMS GQ. From this sample, 209 students agreed to participate in this study. The response rate was 74.6%. The demographic data of the respondents is shown in Table 1. About 66.0% of the respondents were male. Majority of the respondents were in 22–24 (51.2%) and 25–27 (43.5%) age groups. The graduating students were from 13 countries of Asia or Africa. Indian participants (58.4%) outnumbered the participants from all the other countries combined. Six students did the internship in their home countries, while the rest did the internship in China. Data showed that 76.1% of the respondents were satisfied with the overall quality of the medical education.

**Table 1:** Demographic data of the respondents

	University A (n)	University B (n)	University C (n)	University D (n)	Total (n/%)
**Questionnaire distributed**	56	135	69	20	280
**Respondents**	54	112	35	8	209 (100%)
**Gender**					
Male	38	80	14	6	138 (66.0%)
Female	16	32	21	2	71 (34.0%)
**Age upon graduation**					
Invalid ^a^	0	2	1	0	3 (1.4%)
22–24	35	55	15	2	107 (51.2%)
25–27	19	47	19	6	91 (43.5%)
28–30	0	7	0	0	7 (3.4%)
31–33	0	1	0	0	1 (0.5%)
**Country of origin**					
India	38	69	13	2	122 (58.4%)
Nepal	9	36	0	0	45 (21.5%)
Bangladesh	4	5	0	0	9 (4.3%)
Mauritius	0	0	7	0	7 (3.3%)
Sri Lanka	0	0	7	0	7 (3.3%)
Thailand	0	0	6	0	6 (2.9%)
Pakistan	0	2	0	3	5 (2.4%)
Ghana	0	0	1	1	2 (1.0%)
Somalia	2	0	0	0	2 (1.0%)
Afghanistan	0	0	0	1	1 (0.5%)
Comoros	1	0	0	0	1 (0.5%)
Indonesia	0	0	1	0	1 (0.5%)
Jordan	0	0	0	1	1 (0.5%)

Note: ^a^ Three respondents accidentally type their graduation year “2019” instead of their age, so their responses to this question were considered as invalid

### Preparatory and preclinical education experience

**Fig. 1:**
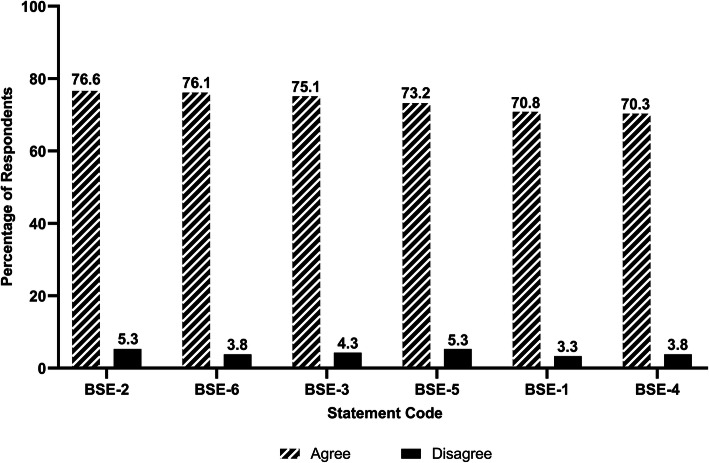
Respondents’ ratings for the statements about basic science education. Statement Code Explanations:BSE-1: Basic science coursework had sufficient illustrations of clinical relevance. BSE-2: Required clinical experiences integrated basic science content. BSE-3: Basic science content objectives and examination content matched closely. BSE-4: Basic science content was sufficiently integrated/coordinated. BSE-5: Basic science content was well organized. BSE-6: Basic science content objectives were made clear to students. Note: n = 209

Figure 1 shows the IMSs’ ratings for the statements about basic science education. Over 70% of the responses were positive in all 6 statements. 76.6% of the students believed that required clinical experiences integrated basic science content, and 76.1% agreed that basic science content objectives were made clear to them. By comparison, 70.3% of the respondents thought basic science content was sufficiently integrated/coordinated, and 70.8% agreed that basic science coursework had sufficient illustrations of clinical relevance.

**Fig. 2:**
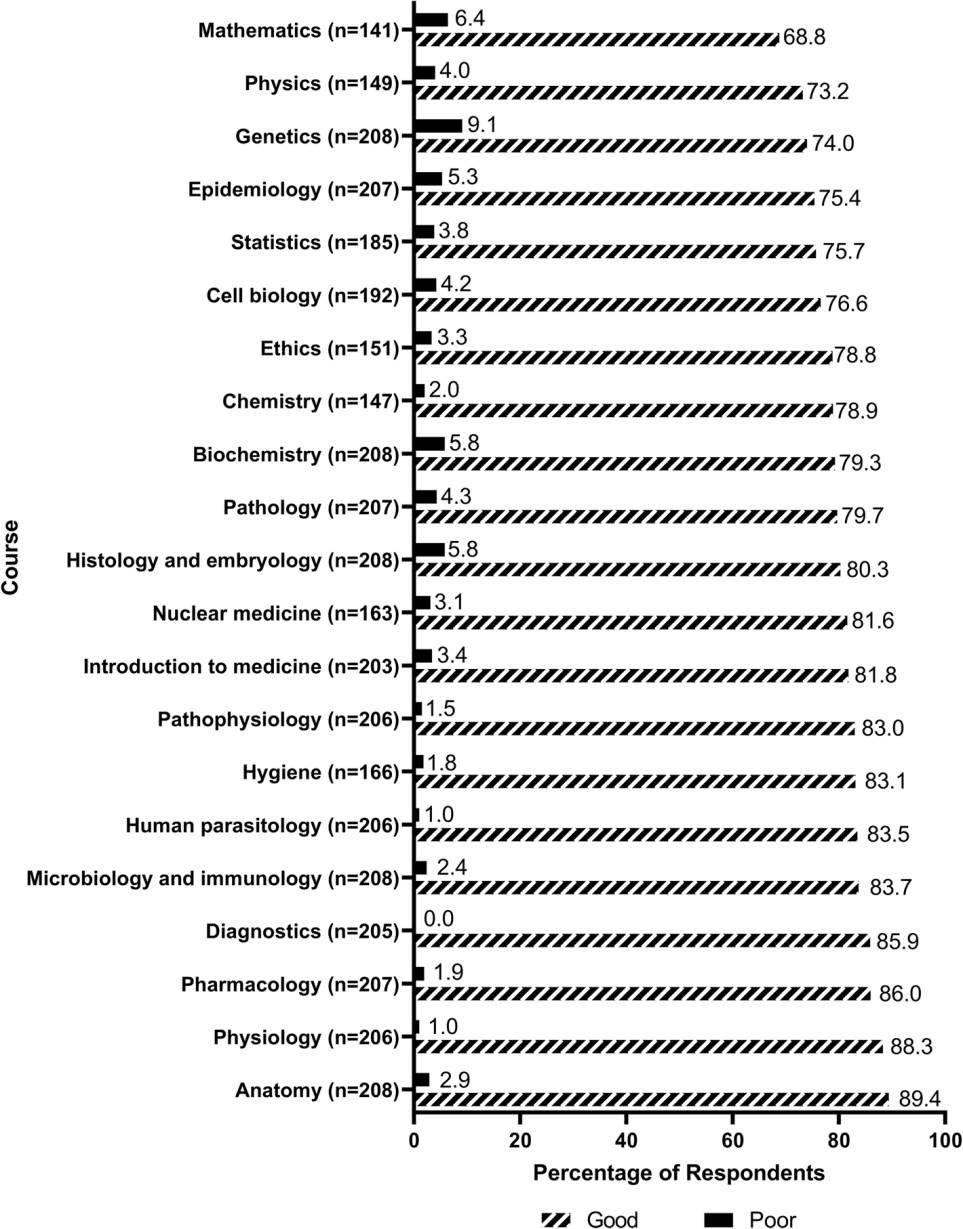
Respondents’ ratings for how the natural science and basic science courses prepare them for clinical clerkships. Notes: For each subject, the choice of “Not applicable” was provided. This choice was to be used if the respondents felt they were not privy to such knowledge. The number of the respondents who chose “Not applicable” for each subject was excluded from the total count for this subject when the percentage was accumulated. The number of the respondents who provided a rating other than “Not applicable” was given after each subject in this figure

Figure 2 shows how the respondents evaluated the specific natural science and basic science courses in preparation for their clinical clerkships. With regards to minor variations in the curricula for IMSs in the 4 participating universities, the choice of “Not applicable” was provided in this section. This choice was to be used if the respondents felt they were not privy to such knowledge. Anatomy (89.4%), physiology (88.3%), and pharmacology (86.0%) were considered as the most helpful courses for their clinical work, while genetics (74.0%), physics (73.2%), and mathematics (68.8%) were considered as the least helpful courses with regards to laying down the foundation for the clerkship.

### Clinical education experience

**Fig. 3:**
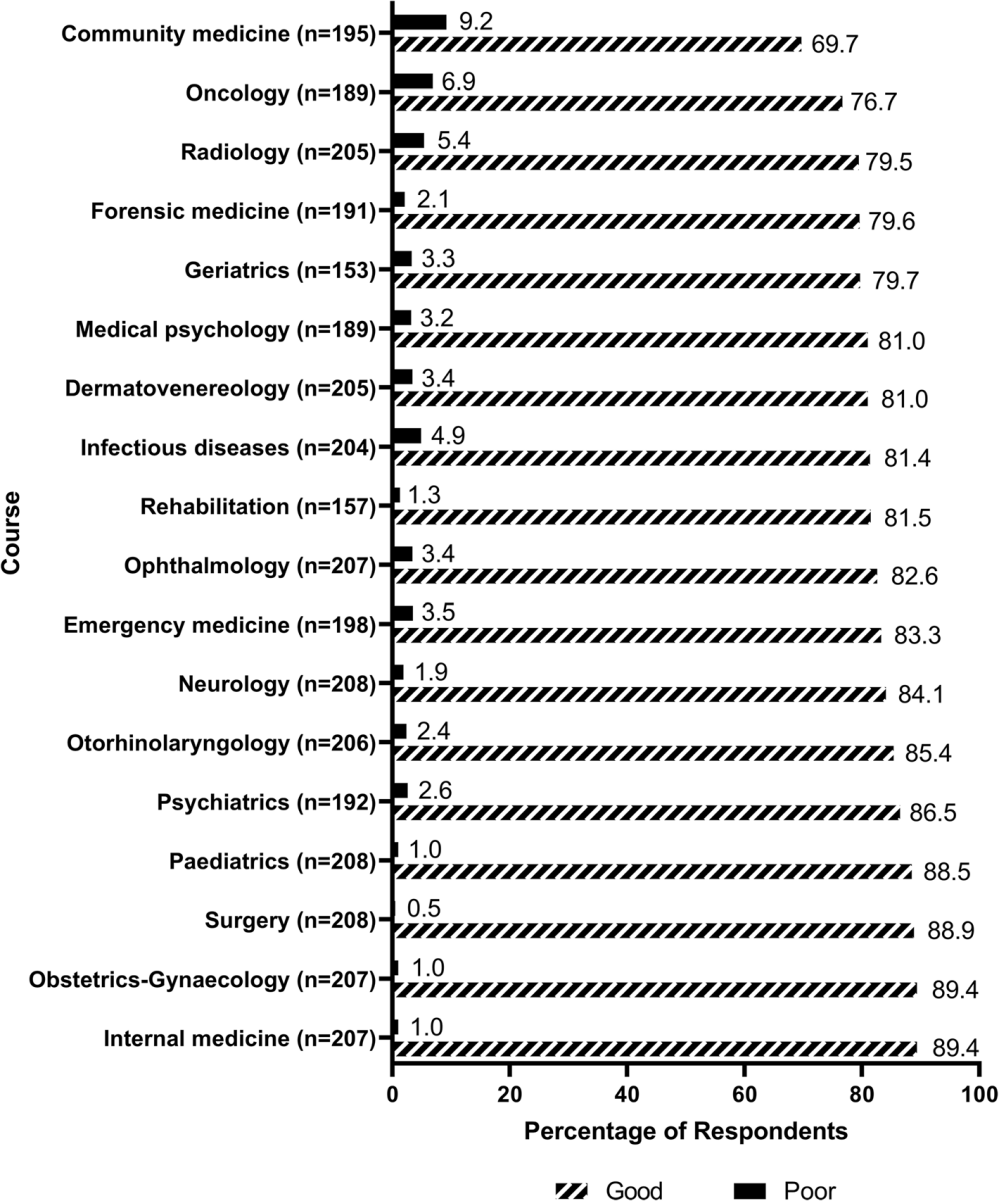
Respondents’ ratings for the quality of their educational experiences in the clinical courses. Notes: For each subject, the choice of “Not applicable” was provided. This choice was to be used if the respondents felt they were not privy to such knowledge. The number of the respondents who chose “Not applicable” for each subject was excluded from the total count for this subject when the percentage was accumulated. The number of the respondents who provided a rating other than “Not applicable” was given after each subject in this figure

Figure 3 shows the respondents’ ratings on their educational experiences in the clinical courses. Internal medicine (89.4%), obstetrics-gynaecology (89.4%), surgery (88.9%) and paediatrics (88.5%) were recognized as good/excellent quality courses by the highest proportion of the respondents, while the satisfactory rate for radiology (79.5%), oncology (76.7%) and community medicine (69.7%) were the lowest.

**Fig. 4:**
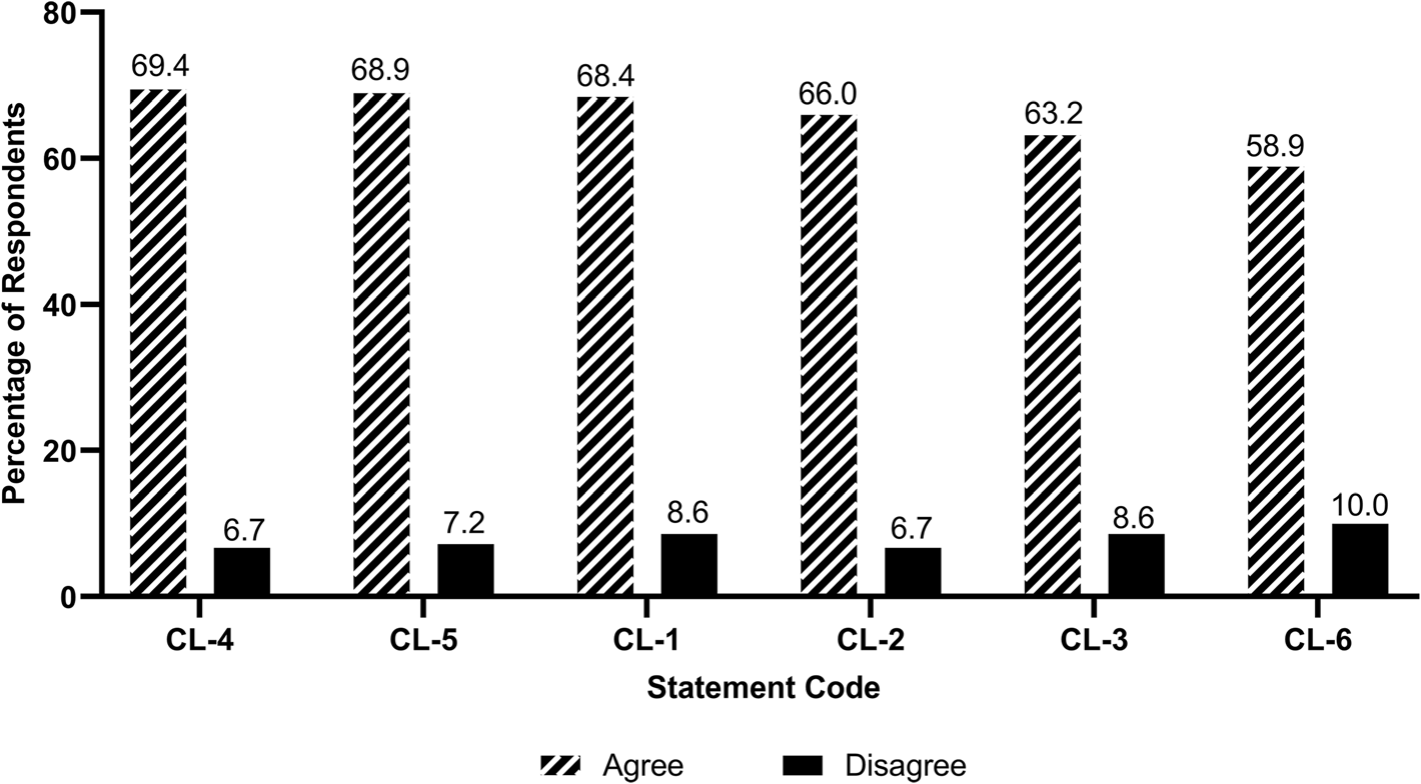
Respondents’ ratings for the statements about clerkship. Statement Code Explanations: CL-1: Faculty provided effective teaching during clerkship. CL-2: The supervision I received was adequate during clerkship. CL-3: There was sufficient use of simulations during the clerkship. CL-4: I had sufficient access to the variety of patients and procedures encountered during clerkship. CL-5: I was given timely feedback on performance in clerkships. CL-6: Ethical issues were discussed during clerkships. Note: *n* = 209

**Fig. 5:**
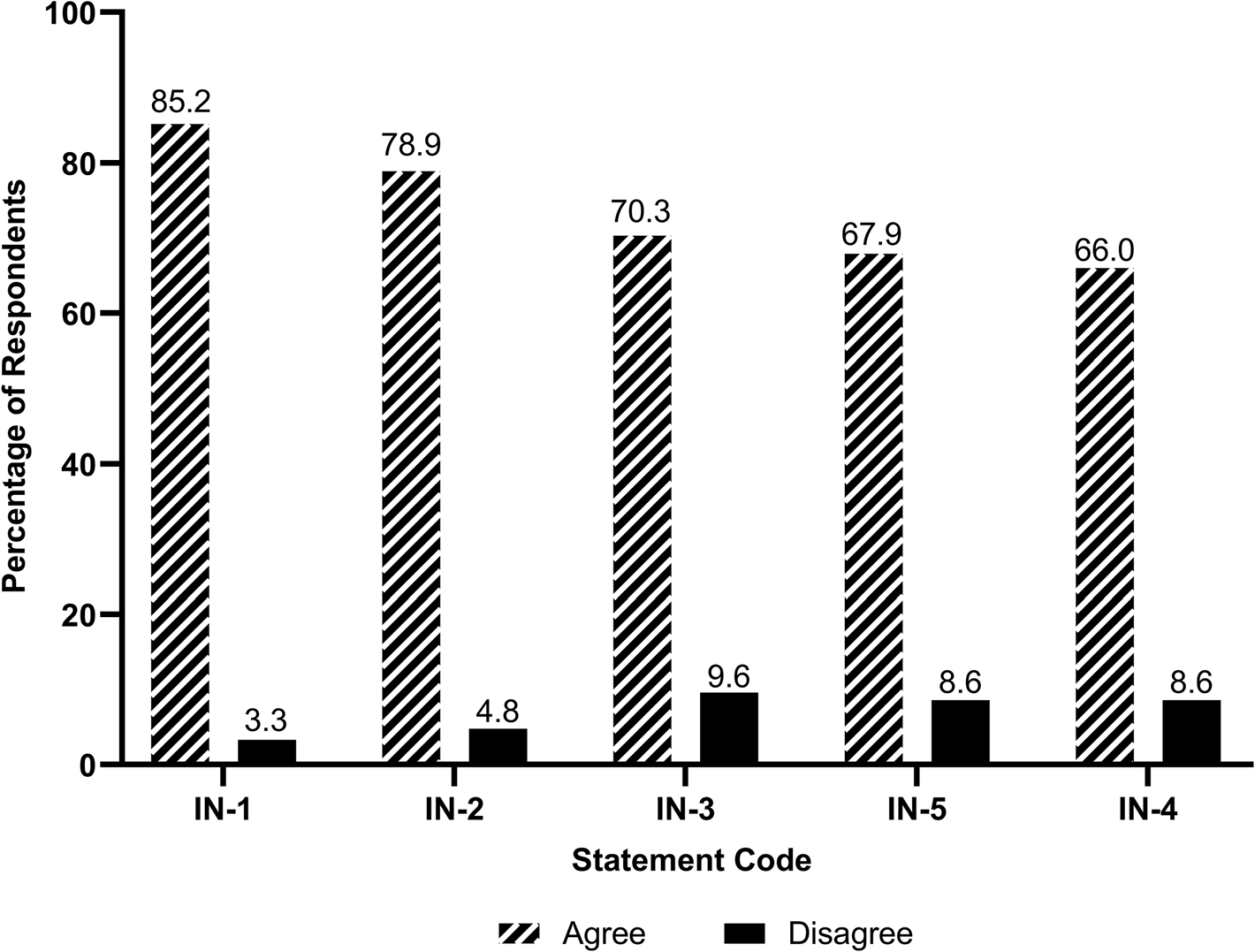
Respondents’ ratings for the statements about internship (in China). Statement Code Explanations: IN-1: The final year (internship) was important for enhancing my medical education. IN-2: The final year (internship) was helpful in my preparations for residency. IN-3: The faculty provided clear guidance on what I needed to learn and do in the internship. IN-4: I was given an appropriate role in patient care during my internship. IN-5: I was taught sufficient clinical skills in preparation for clinical practice as physicians. Note: *n* = 203

Figure 4 shows how respondents rated the statements about clerkship. 69.4% of the students agreed that they had sufficient access to the variety of patients and procedures encountered during clerkship. However, only 58.9% believed that ethical issues were discussed during clerkships. As for internship, although 85.2% of the participants were of the opinion that the final year internship was important for enhancing their medical education, only 66.0% considered that they were given an appropriate role in patient care during the internship (Fig. 5).

**Fig. 6:**
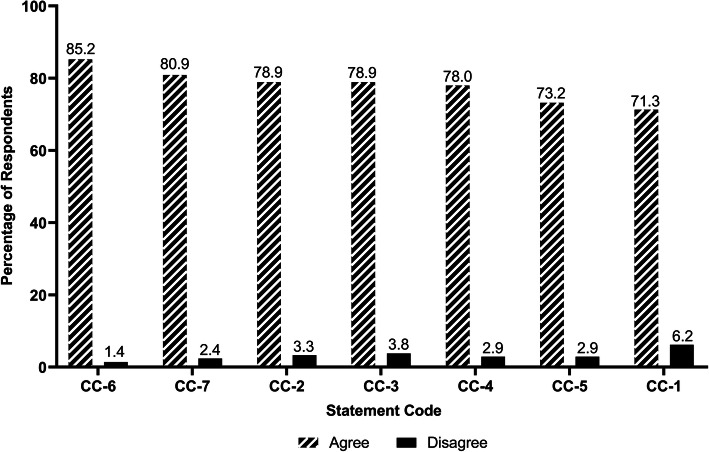
Respondents’ self-evaluation of clinical competence. Statement Code Explanations: CC-1: I am confident that I have acquired the clinical skills required to begin a residency program. CC-2: I have the fundamental understanding of common conditions and their management encountered in the major clinical disciplines. CC-3: I have the communication skills necessary to interact with patients and health professionals. CC-4: I have basic skills in clinical decision making and the application of evidence based information to medical practice. CC-5: I have a fundamental understanding of the issues in social sciences of medicine (e.g., ethics, humanism, professionalism, organization and structure of the health care system). CC-6: I understand the ethical and professional values that are expected of the profession. CC-7: I believe I am adequately prepared to care for patients from different backgrounds. Note: *n* = 209

Regarding clinical competence (Fig. 6), over 80% of the respondents agreed they could understand the ethical and professional values as physicians and were adequately prepared to take care of patients from different backgrounds. However, a comparatively lower rate of students (71.3%) showed confidence in their clinical skills to begin a residency program.

### Benefits of diversity and study hours for specific topics

**Fig. 7:**
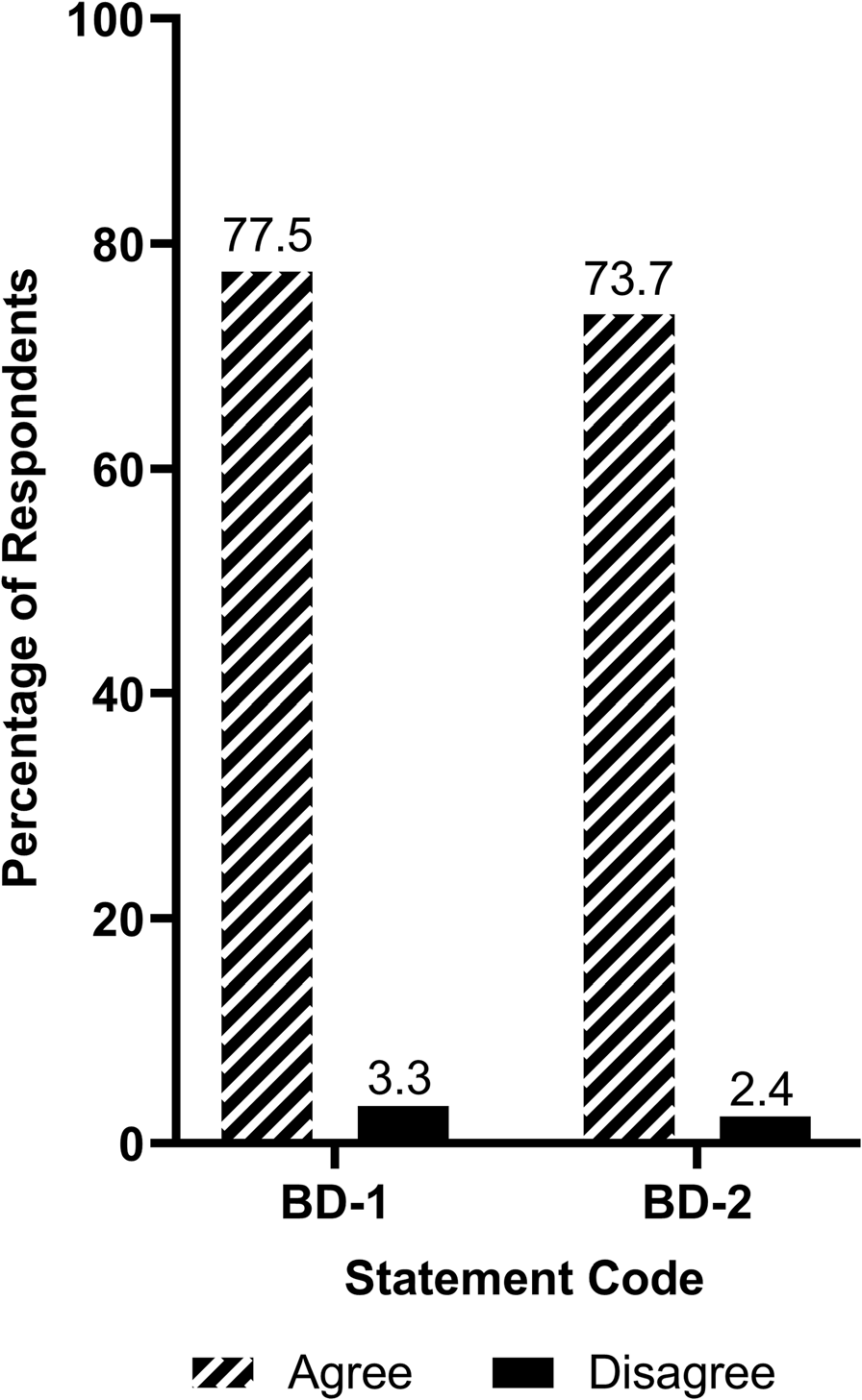
Respondents’ ratings for benefits of diversity. Statement Code Explanations: BD-1: My knowledge or opinion was influenced or changed by becoming more aware of the perspectives of individuals from different backgrounds. BD-2: The cultural diversity within my medical school class enhanced my training and skills to work with individuals from different backgrounds. Note: *n* = 209

**Fig. 8:**
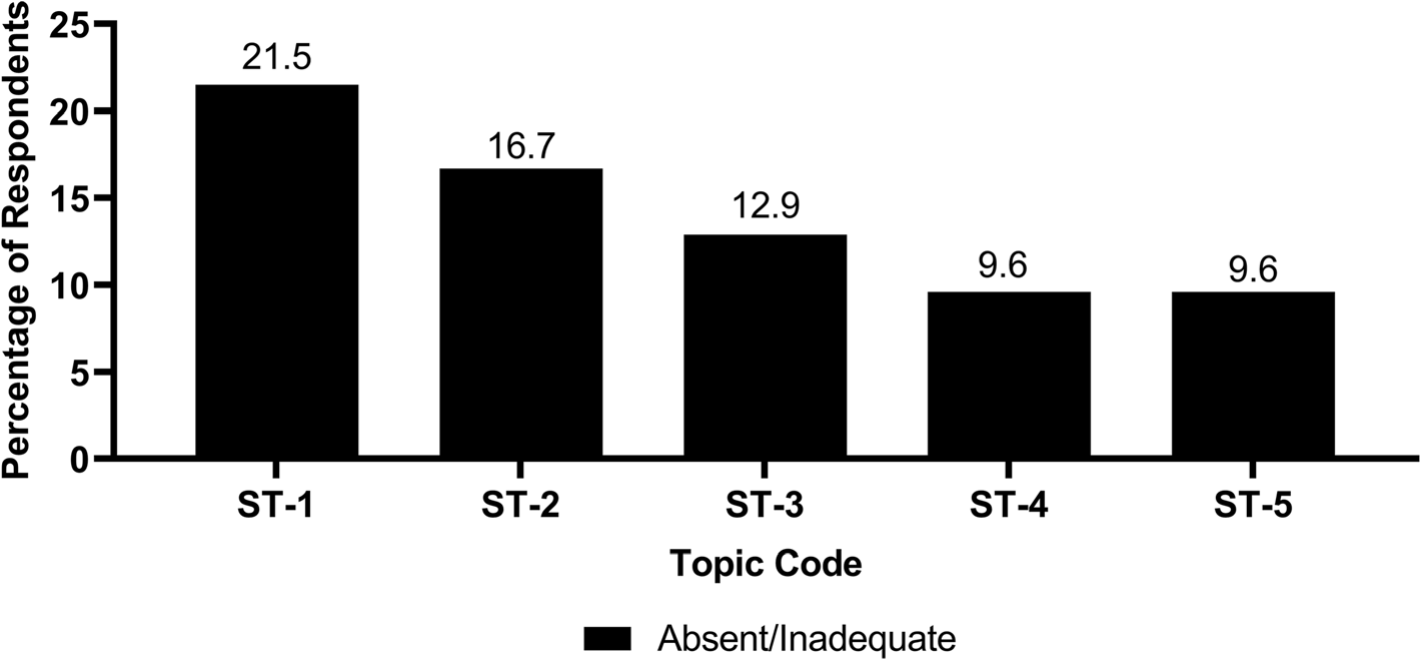
Respondents’ opinions about study hours of specific topics. Topic Code Explanations: ST-1: Community-oriented medicine. ST-2: Culturally appropriate care for diverse populations. ST-3: Practice of medicine. ST-4: Clinical decision making and clinical care. ST-5: Health policy. Note: *n* = 209

Since IMSs are composed of students from diverse cultures and are exposed to teachers and patients from outside their home countries, the benefits of diversity section was included in the survey. It is shown that 73.7% of the respondents thought the cultural diversity in class enhanced their training and skills to work with individuals from different backgrounds (Fig. 7). Regarding study hours for specific topics, community-oriented medicine was voted by a large percentage of the participants (21.5%) as a topic that lacked instruction (Fig. 8).

### Influence of language barrier on clinical studies

Table 2 shows how the respondents’ Chinese language capability influenced their clinical experiences and competence. A total of 18 statements were classified into clerkship, internship and clinical competence fields. HSK level was found to be a significant factor for only 1 statement in the internship field (IN-4). Respondents with lower HSK levels were more likely to believe that they were given an appropriate role in patient care during the internship compared to their counterparts with higher HSK levels.

**Table 2:** Respondents’ Chinese language capability’s influence on their clinical experiences and competence

	HSK certificate (Mean)			Chinese speaking skills (Mean)				Communication initiative with teachers and patients in Chinese in hospital (Mean)		
	Lower HSK level (***n*** = 105)	Higher HSK level (***n*** = 104)	P	Weak (***n*** = 34)	Adequate (***n*** = 105)	Good (***n*** = 70)	P	Not Active (***n*** = 64)	Active (***n*** = 145)	P
**Clerkship (Subtotal)**	22.17	21.63	.309	20.71	21.29	21.90	.000	20.20	22.66	.000
CL-1	3.73	3.58	.145	3.26	3.59	3.94	.000	3.31	3.81	.000
CL-2	3.68	3.64	.757	3.56	3.55	3.87	.014	3.45	3.75	.007
CL-3	3.63	3.57	.571	3.41	3.50	3.84	.004	3.38	3.70	.006
CL-4	3.74	3.72	.836	3.64	3.62	3.94	.016	3.38	3.89	.000
CL-5	3.83	3.62	.066	3.41	3.60	4.06	.000	3.41	3.86	.000
CL-6	3.56	3.51	.657	3.41	3.43	3.76	.018	3.28	3.65	.004
**Internship (Subtotal)**	19.41	19.27	.769	18.53	18.99	20.26	.027	17.98	19.94	.000
IN-1	4.10	4.18	.396	4.18	4.06	4.24	.257	3.98	4.21	.046
IN-2	3.96	4.01	.657	3.91	3.93	4.10	.316	3.77	4.08	.006
IN-3	3.78	3.78	.986	3.50	3.71	4.01	.009	3.48	3.91	.001
IN-4	3.82	3.57	.039	3.41	3.59	3.99	.002	3.36	3.84	.000
IN-5	3.75	3.73	.858	3.53	3.70	3.91	.079	3.39	3.90	.000
**Clinical competence (Subtotal)**	27.03	27.05	.970	26.09	26.54	28.24	.004	25.34	27.79	.000
CC-1	3.75	3.72	.773	3.41	3.69	3.97	.002	3.45	3.86	.000
CC-2	3.87	3.85	.820	3.82	3.76	4.01	.039	3.61	3.97	.000
CC-3	3.91	3.86	.564	3.59	3.87	4.06	.008	3.55	4.03	.000
CC-4	3.90	3.82	.387	3.82	3.74	4.04	.010	3.61	3.97	.000
CC-5	3.76	3.86	.344	3.59	3.74	4.01	.007	3.56	3.92	.001
CC-6	3.90	4.05	.053	3.91	3.92	4.07	.198	3.83	4.03	.016
CC-7	3.94	3.90	.656	3.94	3.82	4.07	.033	3.73	4.01	.004

Chinese speaking skill was a significant factor in 14 of all the 18 statements. The Least Significant Difference (LSD) analysis was performed for pairwise comparisons among the weak, adequate and good Chinese speaking skill groups. Respondents with good Chinese speaking skills had significantly higher scores compared to respondents with weak and/or adequate Chinese speaking skills in the 14 statements. In the CL-1 statement, the adequate group was also found to have significantly higher scores compared to the weak group.

The communication initiative was found to be an absolute influential factor for respondents’ clinical experiences and competence. Respondents with active communication initiative had significantly higher scores compared to those with an inactive communication initiative in all the 18 statements.

## Discussion

This study conducted a graduation survey among IMSs in 4 Chinese universities using a questionnaire referring to AAMC GQ [16] and other graduation surveys [10, 18, 21–23]. We evaluated the effectiveness of the undergraduate program for IMSs in China from multiple aspects and highlighted the influence of language barrier on IMSs’ education experiences. This was conducted to provide useful information for policymakers and university authorities to make important curricular decisions about international medical education for Asian and African students.

Regarding the basic science education, the ratings of IMSs were generally positive, although it still indicates relatively insufficient interdisciplinarity and clinical relevance in the basic science subjects. Studies in India [26] and Nepal [27] also reported similar findings. Similar to China, many Asian and African countries conventionally divide the medical curriculum into 2 separate parts (basic sciences and clinical sciences) and follow discipline-based teaching pattern [26–28]. Consequently, basic sciences are delivered as individual subjects with the least cross-subject interaction or clinical practice integration. To address these weaknesses, problem-based learning (PBL) was introduced for a better integration of the basic medical and clinical contents [29], and it is increasingly popular in Asia and Africa [28, 30–33]. Medical schools in China began to adopt PBL teaching strategy on Chinese medical students since the mid-80s [34], and started to adopt this approach on IMSs more recently with positive results [35–37]. However, it has been reported that the teaching faculties have yet to overcome the language challenge [38].

Among the basic science courses, anatomy and physiology received the highest percentage of agreement in regard to underpinning IMSs’ clerkship. These two subjects also ranked top in other GQs in the USA [16], Canada [18], Iran [22] and Israel [23]. As anatomy, physiology and pharmacology are among the fundamental sciences to medical practice [39], the importance of these subjects is unquestionable. Another possible reason for the high rating is that medical students undergo a significant knowledge loss of basic sciences in their later years of education [40, 41], but pathophysiology-related basic subjects are better remembered [39], because the constant reference to clinical application could reinforce students’ insight into these subjects [42].

In contrast, despite their close relationship with clinical diseases, statistics, epidemiology and genetics were among the least helpful preclinical subjects for medical practice in our survey as well as some other surveys conducted elsewhere [16, 18, 22, 23]. Notably, students need mathematical knowledge to understand statistics and epidemiology. If teachers in China prepare lessons based on the general academic background of Chinese students, who are known for good command of mathematics, there might exist a mismatch between the difficulty of the lessons imparted by teachers and that accepted by IMSs. Genetics is a rapidly advancing discipline involving state-of-the-art concepts and latest research findings, so IMSs will be discontent if the teachers cannot stay abreast of the relevant scientific advances. Furthermore, due to the inadequate application of evidence-based medicine in some countries, IMSs may have not recognized the importance of this subject.

Here, many participants gave negative responses to the benefits of natural sciences for medical practice, a finding supported by evidence from published literature [10, 23]. This is possibly attributed to the poor knowledge retention of the related subjects and a poor integration between natural science and clinical application. However, Goldszmidt et al. [43] argued that using the knowledge of natural science, such as physics, to illustrate clinical phenomena could produce a causal explanation of the latter, which would promote medical students’ memory for clinical details.

For clinical medical education, our findings are in consonance with the reports that the education experience in internal medicine, obstetrics-gynaecology, surgery and paediatrics ranked top 4 among all the clinical courses [10, 21]. Nevertheless, the quality of community medicine was considered the lowest in our study, whereas medical students in surveys conducted in Canada and Iran spoke highly of this subject [18, 22]. Moreover, the large percentage of IMSs who deemed the study hours for community medicine were inadequate further illustrated the participants’ dissatisfaction with this course.

The low rating of IMSs in community medicine could be partially attributed to the large difference in the concepts and contents of this course between China and their countries of origin. In the eyes of IMSs, community medicine is supposed to be a course encompassing all the knowledge and practice related to primary care. However, in China, the primary care contents are integrated into the respective clinical disciplines (e.g. internal medicine, paediatrics, obstetrics-genecology, geriatrics). This is primarily because the health care system in China is hospital-based and the general practitioner based primary care and referral system is highly underdeveloped [44]. This could give IMSs an impression of deficiency in community medicine education. In IMSs’ countries of origin, which are generally medically underserved, the population need to benefit from the enhancement of primary care and an increase in general practitioners [45, 46]. Thus, the medical schools in these countries have been emphasizing community-oriented approaches [28, 47–50], aiming at producing health professionals with competencies and values to serve in local communities, particularly rural areas [51]. Given the vital position of community medicine in IMSs’ home countries, policy planners and educators in China are advised to consider reorienting the related syllabus in line with the need of IMSs, putting more primary care contents into the community medicine course and creating more practice opportunities in a community setting.

Our findings regarding the quality and outcome of clinical education imply that the clinical training of IMSs in China is still insufficient. Specifically, linguistically demanding tasks tend to receive lower ratings. A case in point is that talking ethical topics in depth involves using sophisticated words, and correspondingly, over 40% of our participants provided a negative response towards ethical discussions during their clerkship. Moreover, as patient administration requires a high level of Chinese language proficiency, it was not surprising that a high percentage of our participants complained that they were not assigned an appropriate role in patient care during the internship. Our findings also demonstrate that, IMSs’ speaking skills and communication initiatives, compared to HSK levels, have a greater impact on their clinical experiences and competence. Therefore, medical schools in China are advised to strengthen the oral Chinese teaching for IMSs and encourage students to take initiative in speaking, since doing so will promote a productive time and good education experiences for the students in their clinical clerkship and rotations.

Moreover, IMSs’ clinical study is also influenced by cross-cultural issues. Firstly, although the respondents were quite positive towards the access to various patients and procedures during their clerkship, they had relatively low confidence in the clinical skills they had acquired. In addition to the language barrier, local patients’ strong awareness of self-protection and privacy could present difficulties to IMSs’ hands-on experiences during the process [52]. As a remedy, simulations have been highly recommended by researchers and applied in many medical schools in China as an effective method for clinical teaching for IMSs [52, 53]. Secondly, cross-cultural factors are also influential for ethics teaching, since ethical topics are highly culture-specific, and thus the ethical standards for IMSs are diversified. Therefore, IMSs will not be able to obtain an appropriate and deep understanding of ethical topics without practicing in their home countries or other destination countries.

## Conclusion

In conclusion, we found that there was a lack of integration between basic sciences and clinical practice and a need to reorganize community-oriented coursework in international medical education in China. We also found IMSs with stronger Chinese speaking skills and initiatives were more likely to think highly of the clinical training. These findings indicate that the curriculum for IMSs in China should be more problem-based and community-engaged, and should focus more on oral Chinese teaching. Our findings provide scientific basis for the government and educators to improve the quality of medical curriculum for Asian and African medical students.

## Supplementary Information

**Additional file 1.** Graduation Survey for International Medical Students.

## Data Availability

The datasets used and/or analysed during the current study are available from the corresponding author on reasonable request.

## Abbreviations

AAMC: Association of American Medical Colleges
AFMC: Association of Faculties of Medicine of Canada
BD: Benefits of diversity
BSE: Basic science education
CC: Clinical competence
CL: Clerkship
GQ: Graduation Survey
HSK: Hanyu Shuiping Kaoshi (Chinese language proficiency test)
IMS: International medical student
IN: Internship
PBL: Problem-based learning
ST: Specific topics
UK: United Kingdom
USA: United States of America
