# Supplementary material for: Perceptions of education quality and influence of language barrier: graduation survey of international medical students at four universities in China
Source: BMC Med Educ. 2020 Nov 7;20:410. doi: 10.1186/s12909-020-02340-w (PMC7648950; doi:10.1186/s12909-020-02340-w)
Supplement: Supplementary file 1 — Additional file 1. Graduation Survey for International Medical Students. [file 12909_2020_2340_MOESM1_ESM.docx]

**Graduation Survey for International Medical Students**

This Graduation Survey seeks information from graduating international medical students in China to help medical schools benchmark and improve their medical education programs. The survey asks your opinions about educational programs at your university and your preparation for residency. The information will also be used for research on medical education. Thank you for your voluntary participation.

1. I am a student from:

| Xuzhou Medical University |
| --- |
| Hainan Medical University |
| Dali University |
| Nanjing Medical University |

2. I have read and understood the purpose of this survey and agree to participate. By continuing with this survey I grant permission to share my responses and give consent for my opinions to be published anonymously.

| Yes |
| --- |
| No |

3. Gender

| Male |
| --- |
| Female |

4. Age upon graduation ________

5. Nationality ________

6. I have passed the following Chinese language competence test (Please choose the highest level you have passed):

| HSK 1 |
| --- |
| HSK 2 |
| HSK 3 |
| HSK 4 |
| HSK 5 |
| HSK 6 |
| None |

7. How can you speak/use Chinese in hospital environment?

| Good |
| --- |
| Adequate |
| Weak |

8. Can you actively communicate with teachers and patients in Chinese in hospital?

| Yes |
| --- |
| No |

9. How about your English proficiency?

| Good |
| --- |
| Adequate |
| Weak |

10. Apart from your mother language, can you speak any other languages?

| French |
| --- |
| German |
| Arabic |
| Spanish |
| Others (Please write down the language): |

**Please indicate the extent to which you agree with the following statement:**

11. Overall, I am satisfied with the quality of my medical education.

| Strongly disagree |
| --- |
| Disagree |
| Neutral |
| Agree |
| Strongly agree |

**Basic Science Courses: Based on your experiences, indicate whether you agree or disagree with the following statements about medical school?**

12. Basic science coursework had sufficient illustrations of clinical relevance.

| Strongly disagree |
| --- |
| Disagree |
| Neutral |
| Agree |
| Strongly agree |

13. Required clinical experiences integrated basic science content.

| Strongly disagree |
| --- |
| Disagree |
| Neutral |
| Agree |
| Strongly agree |

14. Basic science content objectives and examination content matched closely.

| Strongly disagree |
| --- |
| Disagree |
| Neutral |
| Agree |
| Strongly agree |

15. Basic science content was sufficiently integrated/coordinated.

| Strongly disagree |
| --- |
| Disagree |
| Neutral |
| Agree |
| Strongly agree |

16. Basic science content was well organized.

| Strongly disagree |
| --- |
| Disagree |
| Neutral |
| Agree |
| Strongly agree |

17. Basic science content objectives were made clear to students.

| Strongly disagree |
| --- |
| Disagree |
| Neutral |
| Agree |
| Strongly agree |

18. Natural and Basic Science Courses: How well did your study of the following sciences basic to medicine prepare you for clinical clerkships? If your medical school does not provide this subject, please choose "Not applicable".

|  | Very poor | Poor | Fair | Good | Excellent | Not applicable |
| --- | --- | --- | --- | --- | --- | --- |
| Biochemistry |  |  |  |  |  |  |
| Epidemiology |  |  |  |  |  |  |
| Genetics |  |  |  |  |  |  |
| Anatomy |  |  |  |  |  |  |
| Microbiology and immunology |  |  |  |  |  |  |
| Introduction to medicine |  |  |  |  |  |  |
| Histology and embryology |  |  |  |  |  |  |
| Physiology |  |  |  |  |  |  |
| Pathology |  |  |  |  |  |  |
| Pharmacology |  |  |  |  |  |  |
| Pathophysiology |  |  |  |  |  |  |
| Cell biology |  |  |  |  |  |  |
| Human parasitology |  |  |  |  |  |  |
| Hygiene |  |  |  |  |  |  |
| Physics |  |  |  |  |  |  |
| Mathematics |  |  |  |  |  |  |
| Chemistry |  |  |  |  |  |  |
| Statistics |  |  |  |  |  |  |
| Diagnostics |  |  |  |  |  |  |
| Ethics |  |  |  |  |  |  |
| Nuclear medicine |  |  |  |  |  |  |

**Clinical experiences**

19. Clinical experiences: Rate the quality of your educational experiences in the following clinical courses. If you participated in an integrated clerkship, please answer this question in terms of your educational experience in each discipline. If you had no clinical experiences in the discipline, select "Not applicable".

|  | Very poor | Poor | Fair | Good | Excellent | Not applicable |
| --- | --- | --- | --- | --- | --- | --- |
| Emergency medicine |  |  |  |  |  |  |
| Internal medicine |  |  |  |  |  |  |
| Neurology |  |  |  |  |  |  |
| Obstetrics- Gynaecology |  |  |  |  |  |  |
| Paediatrics |  |  |  |  |  |  |
| Medical psychology |  |  |  |  |  |  |
| Surgery |  |  |  |  |  |  |
| Radiology |  |  |  |  |  |  |
| Ophthalmology |  |  |  |  |  |  |
| Oncology |  |  |  |  |  |  |
| Forensic medicine |  |  |  |  |  |  |
| Infectious diseases |  |  |  |  |  |  |
| Dermatovenerology |  |  |  |  |  |  |
| Otorhinolaryngology |  |  |  |  |  |  |
| Psychiatrics |  |  |  |  |  |  |
| Rehabilitation |  |  |  |  |  |  |
| Geriatrics |  |  |  |  |  |  |
| Community medicine |  |  |  |  |  |  |

**Clinical experiences: Please indicate whether you agree or disagree with the following statements.**

**Clerkship**

20. Faculty provided effective teaching during clerkship.

| Strongly disagree |
| --- |
| Disagree |
| Neutral |
| Agree |
| Strongly agree |

21. The supervision I received was adequate during clerkship.

| Strongly disagree |
| --- |
| Disagree |
| Neutral |
| Agree |
| Strongly agree |

22. There was sufficient use of simulations during clerkships.

| Strongly disagree |
| --- |
| Disagree |
| Neutral |
| Agree |
| Strongly agree |

23. I had sufficient access to the variety of patients and procedures encountered during clerkship.

| Strongly disagree |
| --- |
| Disagree |
| Neutral |
| Agree |
| Strongly agree |

24. I was given timely feedback on performance in clerkships.

| Strongly disagree |
| --- |
| Disagree |
| Neutral |
| Agree |
| Strongly agree |

25. Ethical issues were discussed during clerkships.

| Strongly disagree |
| --- |
| Disagree |
| Neutral |
| Agree |
| Strongly agree |

**Internship (Last year)**

26. I did my final year internship in:

| China |
| --- |
| Home country |
| Other countries (Please name the country) |

27. The final year (internship) was important for enhancing my medical education.

| Strongly disagree |
| --- |
| Disagree |
| Neutral |
| Agree |
| Strongly agree |

28. The final year (internship) was helpful in my preparations for residency.

| Strongly disagree |
| --- |
| Disagree |
| Neutral |
| Agree |
| Strongly agree |

29. The faculty provided clear guidance on what I needed to learn and do in the internship.

| Strongly disagree |
| --- |
| Disagree |
| Neutral |
| Agree |
| Strongly agree |

30. I was given an appropriate role in patient care during my internship.

| Strongly disagree |
| --- |
| Disagree |
| Neutral |
| Agree |
| Strongly agree |

31. I was taught sufficient clinical skills in preparation for clinical practice as physicians.

| Strongly disagree |
| --- |
| Disagree |
| Neutral |
| Agree |
| Strongly agree |

**Self-evaluation of preparedness for residency program: Indicate whether you agree or disagree with the following statements about your preparedness for beginning a residency program.**

32. I am confident that I have acquired the clinical skills required to begin a residency program.

| Strongly disagree |
| --- |
| Disagree |
| Neutral |
| Agree |
| Strongly agree |

33. I have the fundamental understanding of common conditions and their management encountered in the major clinical disciplines.

| Strongly disagree |
| --- |
| Disagree |
| Neutral |
| Agree |
| Strongly agree |

34. I have the communication skills necessary to interact with patients and health professionals.

| Strongly disagree |
| --- |
| Disagree |
| Neutral |
| Agree |
| Strongly agree |

35. I have basic skills in clinical decision making and the application of evidence based information to medical practice.

| Strongly disagree |
| --- |
| Disagree |
| Neutral |
| Agree |
| Strongly agree |

36. I have a fundamental understanding of the issues in social sciences of medicine (e.g., ethics, humanism, professionalism, organization and structure of the health care system).

| Strongly disagree |
| --- |
| Disagree |
| Neutral |
| Agree |
| Strongly agree |

37. I understand the ethical and professional values that are expected of the profession.

| Strongly disagree |
| --- |
| Disagree |
| Neutral |
| Agree |
| Strongly agree |

38. I believe I am adequately prepared to care for patients from different backgrounds.

| Strongly disagree |
| --- |
| Disagree |
| Neutral |
| Agree |
| Strongly agree |

**Benefits of diversity: Based on your experiences, indicate whether you agree or disagree with the following statements**

39. My knowledge or opinion was influenced or changed by becoming more aware of the perspectives of individuals from different backgrounds.

| Strongly disagree |
| --- |
| Disagree |
| Neutral |
| Agree |
| Strongly agree |

40. The cultural diversity within my medical school class enhanced my training and skills to work with individuals from different backgrounds.

| Strongly disagree |
| --- |
| Disagree |
| Neutral |
| Agree |
| Strongly agree |

**Time allocation to specific medical issues: do you believe that your instruction in the following areas was inadequate, appropriate or excessive?**

41. Clinical decision making and clinical care

| Absent |
| --- |
| Inadequate |
| Adequate |
| Excessive |

42. Practice of medicine

| Absent |
| --- |
| Inadequate |
| Adequate |
| Excessive |

43. Community-oriented medicine

| Absent |
| --- |
| Inadequate |
| Adequate |
| Excessive |

44. Culturally appropriate care for diverse populations

| Absent |
| --- |
| Inadequate |
| Adequate |
| Excessive |

45. Health policy

| Absent |
| --- |
| Inadequate |
| Adequate |
| Excessive |
